# Consequences of International Sanctions on Iranian Scientists and the Basis of Science

**DOI:** 10.5812/hepatmon.14843

**Published:** 2013-09-20

**Authors:** Soodabeh Saeidnia, Mohammad Abdollahi

**Affiliations:** 1 Medicinal Plants Research Center, Faculty of Pharmacy, Tehran University of Medical Sciences, Tehran, IR Iran; 2 College of Pharmacy and Nutrition, University of Saskatchewan, Saskatoon, Canada; 3 Department of Toxicology and Pharmacology, Faculty of Pharmacy and Pharmaceutical Sciences Research Center, Tehran University of Medical Sciences, Tehran, IR Iran

**Keywords:** Iran, Sanction, Science

*Dear Editor,*

Since 1979 when revolution happened in Iran, this country started facing some embargos and limitations form the US side that step by step became expanded (1). Saying they have focused to Iran’s nuclear program (a peaceful activity to provide fuel of power industries and radio drugs claimed by the country), sanctions have been expanded to investments in oil, gas and petrochemicals, exports of refined petroleum products, and business, banking and insurance transactions, shipping, web-hosting services for commercial endeavors, and domain name registration services (2). The United Nation (UN) and non-UN-mandated sanctions such as the European Union restrictions includes prohibition of collaboration with Iran in the overseas trade, fiscal services, energy sectors and technologies, and forbidding the provision of insurance and reinsurance by insurers in the member states to Iran and Iranian-owned companies (3).

Nevertheless there are disagreement on “What is the extent of the impact of sanctions?” there is no doubt that above-mentioned embargoes could impact on political relations, economy, oil price, humanities and foreign exposure and transactions of Iran. Although pharmaceuticals and medical equipment did not stand under international embargos, the country has faced reduction of medicines for treatment of several diseases such as cancer, cardiovascular and respiratory illnesses, thalassemia and multiple sclerosis (MS). A principle reason is inability of Iran to pay via international transferring systems due to sanctions (4). In a recent contribution, it is reported that pharmaceutical market confirms that the sanctions are affecting ordinary citizens and national health sector resulted in reduction of affordability of patients’ lifesaving medicines (5).

Unfortunately, alongside the above mentioned embargos, there are hidden aspects of such thinking, which directly or indirectly affect the ordinary scientists, academics, researchers and also students inside or outside of the country. For instance, very recently the famous publisher Elsevier inquired its American editors and reviewers not to exert the manuscripts, in which there are any Iranian co-authors employing in the government of Iran (6). This was just following a recent policy persuaded by the US Office of Foreign Assets Control (OFAC; on March 2013), indicating that in order to extend the embargos inflicted on Iran due to its chasing on nuclear power. According to that rule, other companies not originated in the USA have to stop their American staff not to be interacted with the government of Iran (7). Considering this rule, since universities and academic are somehow related to the government in terms of regulations, rules, budgets, accepting students, etc., the rule may affect all scientists and students. However, there has been a discussion by the Committee on Publication Ethics (COPE) on the USA embargoes against Iran and its probable impact on Iranian researchers as well as publishers. COPE reached to the following conclusion to amend code of conducts in publishing as follows: “Editorial decisions should not be affected by the origins of the manuscript, including the nationality, ethnicity, political beliefs, race, or religion of the authors. Decisions to edit and publish should not be determined by the policies of governments or other agencies outside of the journal itself” (8). The important point of the story is that OFAC sanctions have resulted in misunderstanding of some editors in other countries denying handling Iranian manuscripts because of political not scientific reasons (9).

As recently published in BMJ, several papers have been refused by journals in the US and Australia e.g. American Journal of Cardiology (published by Elsevier), the Journal of Ocular Pharmacology and Therapeutics (published in the US by Marie Ann Liebert), and the Australian journal Ophthalmic Epidemiology) in the recent months because of the sanctions. On the other hand, Dove Medical Press (an open access online publisher whose headquarters are in the UK and editorial office is in New Zealand) turned down a paper saying that “international sanctions currently in place against Iran mean that Dove Medical Press is no longer able to process manuscripts received from Iranian authors” (10).

Together with all the mentioned consequences, academic people of the country are facing other side effects of such sanctions during the past years. For example, they have many problems with money transaction in order to do scientific activities for example payment of publication fees, society subscription fee, or registration fee in international congresses, etc. This is due to the reason that Iran is deprived to go through the international payment systems. Boring process of getting visa for participating in academic events, international conferences and symposiums are just the instance examples of the terrible concerns for Iranian faculties and students. The students suffer problems in getting visa from the US and those who study abroad cannot receive their financial supports from their parents inside the country. These all have made extra burden to Iranian scientists and students. Traditionally, the US has been one of the goal countries to which talented Iranian students and scientists are interested to travel to achieve higher scientific positions. At the moment, the challenges of travel between these two countries for scientific collaboration are very daunting. Nonetheless, it seems that there is a silent and progressive collaboration mutually between Iranian and western (especially USA) researchers and faculty members in case of international cooperation on science and technology. The science of “Toxicology” is one of the examples that is growing fast to be global, in which there are diverse countries found in SCImago for their scientific production in toxicology. Surprisingly among 10 top countries Iran from Middle East and USA from Northern America are listed while toxicology societies play a considerable role in such cooperation in presence of all embargos (11).

Although there was a big achievement in science and technology for Iran during the past years (12), the rate of brain drain was not small, which is at least in part attributed to the scientific embargos against Iran regarding to the many troubles occurred for them after expanded sanctions. With no doubt, most of Iranian higher educated people and scientists have a good chance to find considerable jobs in developed countries especially in the US, Canada and European countries (13).

Unfortunately, growing tensions over Iran’s nuclear program consequently affected Iranian academics and students who were going to get visa to visit abroad. It is reported that “at least half a dozen Iranian graduate students in engineering who were slated to attend universities such as Michigan State, Southern Illinois, South Carolina, Pittsburgh and Northwestern this year couldn’t come because the U.S. rejected their visa applications under the same espionage clause”, Bloomberg News has found (14).

Taking together, as the scientist, we believe the sanctions if imposed against any country even intended or unintended hurt the scientists and the basis of science and thus it is an unfair action. Alongside diverse influences of embargoes on sanctioned countries, high graduated people face various problems which can provide many tribulations for them leading to stop or suppress their scientific works, researches and achievements. This might easily interfere in scientific findings not to be spread and published on time or at all leading to delay in reaching to important and key data.
